# Exploring near-infrared spectroscopy and hyperspectral imaging as novel characterization methods for anaerobic gut fungi

**DOI:** 10.1093/femsmc/xtae025

**Published:** 2024-09-10

**Authors:** Markus Neurauter, Julia M Vinzelj, Sophia F A Strobl, Christoph Kappacher, Tobias Schlappack, Jovan Badzoka, Matthias Rainer, Christian W Huck, Sabine M Podmirseg

**Affiliations:** Department of Microbiology, Universität Innsbruck, Technikerstraße 25d, 6020 Innsbruck, Austria; Department of Microbiology, Universität Innsbruck, Technikerstraße 25d, 6020 Innsbruck, Austria; Department of Microbiology, Universität Innsbruck, Technikerstraße 25d, 6020 Innsbruck, Austria; Institute of Analytical Chemistry and Radiochemistry, CCB-Center for Chemistry and Biomedicine, Universität Innsbruck, Innrain 80-82, 6020 Innsbruck, Austria; Institute of Analytical Chemistry and Radiochemistry, CCB-Center for Chemistry and Biomedicine, Universität Innsbruck, Innrain 80-82, 6020 Innsbruck, Austria; Institute of Analytical Chemistry and Radiochemistry, CCB-Center for Chemistry and Biomedicine, Universität Innsbruck, Innrain 80-82, 6020 Innsbruck, Austria; Institute of Analytical Chemistry and Radiochemistry, CCB-Center for Chemistry and Biomedicine, Universität Innsbruck, Innrain 80-82, 6020 Innsbruck, Austria; Institute of Analytical Chemistry and Radiochemistry, CCB-Center for Chemistry and Biomedicine, Universität Innsbruck, Innrain 80-82, 6020 Innsbruck, Austria; Department of Microbiology, Universität Innsbruck, Technikerstraße 25d, 6020 Innsbruck, Austria

**Keywords:** Neocallimastigomycota, strain characterization, mid-infrared spectroscopy, near-infrared spectroscopy, alternative strain discrimination

## Abstract

Neocallimastigomycota are a phylum of anaerobic gut fungi (AGF) that inhabit the gastrointestinal tract of herbivores and play a pivotal role in plant matter degradation. Their identification and characterization with marker gene regions has long been hampered due to the high inter- and intraspecies length variability in the commonly used fungal marker gene region internal transcribed spacer (ITS). While recent research has improved methodology (i.e. switch to LSU D2 as marker region), molecular methods will always introduce bias through nucleic acid extraction or PCR amplification. Here, near-infrared spectroscopy (NIRS) and hyperspectral imaging (HSI) are introduced as two nucleic acid sequence-independent tools for the characterization and identification of AGF strains. We present a proof-of-concept for both, achieving an independent prediction accuracy of above 95% for models based on discriminant analysis trained with samples of three different genera. We further demonstrated the robustness of the NIRS model by testing it on cultures of different growth times. Overall, NIRS provides a simple, reliable, and nondestructive approach for AGF classification, independent of molecular approaches. The HSI method provides further advantages by requiring less biomass and adding spatial information, a valuable feature if this method is extended to mixed cultures or environmental samples in the future.

## Teaser

Near-infrared spectroscopy and hyper-spectral imaging are explored as novel methods for characterization of anaerobic gut fungi strains, independent of molecular approaches.

## Introduction

Anaerobic gut fungi (AGF) are an early branching lineage of fungi (phylum Neocallimastigomycota) that inhabit the digestive tract of herbivores. These unique fungi have evolved to thrive in the oxygen-deprived milieu of their host’s digestive system, where they act as crucial symbionts in the intricate process of plant biomass degradation and nutrient cycling (Gruninger et al. 2014, Hess et al. 2020). They possess a unique set of potent enzymes to breakdown lignocellulosic plant material, making them promising candidates for biotechnological applications, such as bioethanol production, generation of biomolecules, or biogas production from organic wastes (Dollhofer et al. 2015, Yildirim et al. 2017, Vinzelj et al. 2020, Liu et al. 2021).

AGF display a complex life cycle, switching between a nonmotile vegetative state and a mobile zoospore phase. Their flagellated zoospores attach to plant material, where they encyst and form either a filamentous or a bulbous thallus that penetrates and grows through the plant substrate. Reproduction in AGF occurs presumably asexually, via formation of one or more sporangia that release new motile zoospores upon maturation, thus completing the life cycle. For species with monocentric, filamentous thalli, anucleate rhizoids are formed, where the nucleus remains in the cyst and only one sporangium is formed. For species with polycentric, filamentous thalli, nuclei migrate through the rhizoidal system, which may lead to the formation of multiple sporangia. Species with bulbous thallus development produce spherical holdfasts for the penetration of plant matter (Theodorou et al. 1996, Gruninger et al. 2014, Hanafy et al. 2022).

While having been described and isolated nearly 50 years ago (Orpin 1975), AGF are still poorly understood. This is mainly due to challenges in their isolation and their complex growth requirements (Vinzelj et al. 2022, Meili et al. 2023). Further, genome-based studies on AGF are hampered by their large and repetitive genomes with high AT-content (up to 83%), impeding standard sequencing approaches (Youssef et al. 2013, Edwards et al. 2017, Meili et al. 2023). There are currently 22 described genera of AGF (Hanafy et al. 2022, Pratt et al. 2023) and culture-independent techniques suggest the existence of even more than double this amount (Paul et al. 2018, Meili et al. 2023). The internal transcribed spacer 1 (ITS1) is a region commonly used to identify fungal species and is also frequently used as a barcode for AGF (Schoch et al. 2012, Edwards et al. 2019, Hess 2020 et al. 2020). However, this region exhibits significant length heterogeneity within AGF strains (Edwards et al. 2008), with variation of up to 13% from clones within a single culture (Callaghan et al. 2015). This makes sequence alignment challenging and poses problems to ITS1 based phylogeny (Edwards et al. 2017, 2019, Hanafy et al. 2020). While ITS2 is also a standard region for fungal taxonomy, it is less common than ITS1 for AGF. More recently, the usage of the 28S large ribosomal subunit (LSU), (D1/D2 domain) was broadly adopted by the AGF research community for phylogenetic and taxonomic analyses. This region contains negligible intrastrain sequence divergence and displays fewer length heterogeneities (Hanafy et al. 2020, Elshahed et al. 2022). LSU-based taxonomy has led to the construction of a well-resolved phylogenetic tree, still with unresolved taxonomic issues at the family level due to lack of genomic data (Hanafy et al. 2023). Overall, the challenges posed by the unique genomic features of AGF and struggles with commonly used fungal barcodes (Schoch et al. 2012, Edwards et al. 2017) make alternative identification and characterization methods of high interest.

Apart from the genomic sequence of an organism, the relative abundances of biomolecules in its cells, whether they are used for cell-structure, metabolism, or energy storage, can provide insights into the unique biochemical composition of an organism. Hence, the chemical configuration of cells can be used for discrimination of strains. This is where analytical techniques, such as mass spectrometry and infrared spectroscopy, expand our knowledge of species identification via a more wholistic cellular characterization on top of standard DNA-based approaches (Ngo-Thi et al. 2003, Erukhimovitch et al. 2005, Krásný et al. 2013, Chalupová et al. 2014).

In recent decades, near-infrared spectroscopy (NIRS) has emerged as a powerful tool to discriminate between different bacterial strains, even down to the subspecies level (Cámara-Martos et al. 2012, Quintelas et al. 2018, Tian et al. 2021). NIRS involves the measurement of light absorption in the near-infrared region of the electromagnetic spectrum (750–2500 nm). This technique exploits the overtone and combination vibrations of various molecular bonds, giving broad absorption bands. This results in a so-called “spectral fingerprint” of each sample. Variations in key biomolecules, including proteins, lipids, and carbohydrates, can be detected with NIRS and conclusions about their relative abundance and hence the composition of the sample can be drawn. Further, through chemometrics and multivariate data-analyses, these variations can be used to correlate samples with certain spectra and thereby classify unknown samples to a reference strain. NIRS is further advantageous for biological samples as it is nondestructive and requires minimal sample preparation. This enables further downstream analysis of samples, such as nucleic acid extraction, mass spectrometry, or biochemical characterization (Burns and Ciurczak 2008, Pasquini 2018, Ozaki et al. 2021).

The implementation of NIR for identification of filamentous fungi is less established and fungal studies generally use visible light in combination with NIR or hyperspectral imaging (HSI) approaches to characterize strains (Petisco et al. 2008, Piekarczyk et al. 2019, Lu et al. 2020). Overall, the application of NIRS on whole biomass of fungi poses a valuable alternative discrimination method, independent of standard molecular approaches, complementary to the already common practice of MALDI-TOF MS (Hendrickx 2017, Gómez-Velásquez et al. 2021).

HSI—a technique that combines features of light microscopy and infrared spectroscopy (Khan et al. 2018, Wang et al. 2018)—can yield further insight into the spatial composition of samples. Thereby, spectral information on specific colonies of microorganisms or distinct features of fungal thalli, with resolutions up to a few µm are feasible (Gowen et al. 2015, Soni et al. 2022). HSI is currently used to answer complex research questions that require spatial resolution, such as detecting food contamination (Soni et al. 2022) or differentiating developmental stages of fungal growth on agar plates (Lu et al. 2020). For samples with little available biomass, mid-infrared (MIR) light (2.5–25 µm) could prove advantageous over NIR, as absorption is generally much stronger in this region. This is due to the lower energy of MIR light, which excites the more commonly occurring fundamental vibration modes within molecules. These fundamental excitations can be assigned to certain molecular bonds and hence, as compared to the combination and overtone bands in NIR, also functional groups are identified more easily (Pasquini 2018, Beć et al. 2022).

In this study, based on the intriguing insights that the NIRS and HSI methods can offer when inspecting biological samples, and to expand our discrimination efforts beyond the standard molecular approaches for AGF, we had the three following aims: (i) NIR classification: whether NIRS is a valid technique to differentiate among AGF strains and further, if this is possible, which functional groups (and hence which biomolecules) are responsible for this discrimination; (ii) culture age analysis and model extension: if it is possible to detect changes in biomass composition of fungal cultures over time and whether fungal strains harvested at different cultivation times could still be differentiated by a NIRS model; (iii) HSI: lastly, we wanted to test HSI as a tool for discrimination of AGF strains and evaluate the suitability to identify morphologically different structures within a strain.

## Materials and methods

### Strain cultivation and independent characterization

Three pure cultures of AGF strains were used for differentiation in these experiments. They were chosen to serve as reference for different growth characteristics of AGF: *Anaeromyces mucronatus* (polycentric, filamentous growth; sequence accession number: ON614226–ON614231), *Caecomyces communis* (monocentric, bulbous growth; sequence accession number: OP216660), and *Pecoramyces ruminantium* (monocentric, filamentous growth; strain C1A sequence accession number: JN939127; Youssef et al. 2013). Each strain was identified by microscopic inspection (images displayed in appendix Supplementary Fig. 9) and sequencing of the D1/D2 region of the LSU using the GGNL1F and GGNL4R primer pair (Nagler et al. 2018). The absence of commonly co-occurring methanogens was confirmed by brightfield- and fluorescence microscopy (Co-Factor F_420_) and PCR against the V4 region of 16S RNA with the 515f and 806r primer pair (Caporaso et al. 2011). Further, no methane production was detected with gas composition measurement according to Wunderer et al. (2022) (GC-2010, Shimadzu). Defined media (omission of clarified rumen fluid, tryptone, and yeast extract; Strobl et al. 2024) media, containing no antibiotics was used for AGF biomass generation. Briefly, 150 ml of salt solution 1 and 150 ml of salt solution 2 were combined and 2 g of xylan (from beech wood, Carl Roth), 2 ml of resazurin, 2 ml of hemin, and 700 ml of distilled water were added. The solution was heated up close to the boiling point and was then cooled under pure CO_2_ flux. Then, 6 g of NaHCO_3_, 3 g of cellobiose (Carl Roth), 10 ml of trace element solution, and 1 g of l-cysteine HCl were added, and pH was adjusted to 6.9 with NaHCO_3_. A volume of 0.01 ml of vitamin solution per 1 ml of medium was added just before inoculation. Anoxic serum bottles containing 45 ml medium were inoculated with 5 ml of well-growing, 1-week-old fungal cultures from the AGF culture collection at the Department of Microbiology at Universität Innsbruck.

### NIR measurements

#### Sample preparation

30 pure cultures per strain were grown for 7 days in 50 ml defined medium (total *n* = 90) at 39°C in anoxic serum bottles (henceforth, this group will be referred to as “1 w”). Samples were then centrifuged (12 000 rcf for 4 min) and the supernatant was discarded. Subsequently, the fungal biomass was washed thrice with 30 ml deionized water, discarding the supernatant after each centrifugation step. Samples were then lyophilized (VaCo 2, Zirbus, Bad Grund, Germany). The obtained fungal powder was homogenized with a spatula before being filled into quartz reflectance cuvettes for measurement and packed with a metal cylinder. As not all strains had produced enough biomass for downstream analysis an overall sample number of 78 was achieved [*P. ruminantium* (*n* = 25), *C. communis* (*n* = 25), and *A. mucronatus* (n = 28)]. For the culture age analysis and model extension (ii) additional biomass samples, harvested at different time points after inoculation were generated. Three samples of each strain were taken at 72 h after inoculation, as preliminary experiments demonstrated the highest zoospore density for this timepoint (this group will be referred to as “72 h”). For 72 h samples, larger bottles containing 100 ml of medium were used to generate sufficient biomass after shorter growth time. Additionally old cultures were taken [*P. ruminantium* (*n* = 6), *C. communis* (*n* = 3), *A. mucronatus* (*n* = 8)] that had been left at 39°C for over 3 weeks, up to 3 months (this group will be referred to as “≥3 w”). Samples of the 72 h and ≥3 w group were treated as described above prior to measurement.

#### Measurement

Samples were measured with the Büchi NIRFlex N-500 (Büchi Labortechnik AG, Flawil, Switzerland) using the Solids XL top piece. Each sample was measured in triplicate using reflectance mode and 32 scans per measurement. Spectra were obtained at a wavelength range of 4000–10 000 cm^−1^ and a resolution of 4 cm^−1^. The chitin standard (chitin from shrimp cells, Sigma Aldrich) was measured identically to the lyophilized samples.

### HSI

#### Sample preparation

Three biological replicates of pure cultures of each strain were grown for 72 h in 50 ml defined medium (total *n* = 9). For the HSI approach, a shorter cultivation period was used to prevent formation of aggregates in fungal cultures, as this could lead to considerable overlay of morphologically different structures during microscopy (see Supplementary Fig. 9B). Twice, 2 ml of culture were withdrawn from each bottle via a syringe and centrifuged at 500 rcf for 5 min, discarding the supernatant. Centrifugation speed was lowered for this approach, compared to NIRS sample preparation, to preserve the morphological integrity of the strains. Then samples were washed with 1 ml of 1x PBS solution, centrifuged as before, and the supernatant was discarded. The harvested cells were then resuspended in 250 µl of a 1:1 solution of 96% ethanol and 1x PBS solution. Two replicates of each suspended cell solution (each 10 µl) were put on CaF_2_-plates for imaging analysis and dried at room temperature. The three biological replicates, together with two technical replicates from each culture bottle and the duplicate measurement resulted in 12 plates per strain. Before measurement, CaF_2_-plates were dried by an ethanol series (50%, 80%, and 96%) and lastly, samples were dried at 50°C for 30 min. The ethanol series had the additional effect of removing remaining salt from the ethanol/PBS washing step.

#### Measurement

Due to the low absorption intensities in the NIR-region, MIR was chosen as appropriate wavelength region for fungal specimen with only a few µm thickness (Pasquini 2018, Ozaki et al. 2021). Imaging was carried out using the PerkinElmer Spotlight 400 FT-IR Imaging System and the PerkinElmer Spectrum 400 FT-IR/FT-NIR Spectrometer (PerkinElmer Inc., Waltham, MA, USA). Transmission mode was employed for measurement. The wavelength range used was 1000–4000 cm^−1^ with a resolution of 4 cm^−1^. A pixel size of 6.25 µm was chosen with 16 scans per pixel. For each plate, a minimum of three different cell structures with thickness, leading to sufficient absorption of MIR light, were selected, resulting in 156 total measurements [*P. ruminantium* (*n* = 59), *C. communis* (*n* = 47), and *A. mucronatus* (*n* = 48)]. Additionally, for *P. ruminantium* 39 spectra of sporangia were collected, as this strain exhibited most characteristic, morphological differences between hyphae and sporangia.

### Data processing

#### NIR

All spectra were processed using The Unscrambler X (Version 10.5, CAMO, Oslo, Norway). For NIR, the three technical replicate measurements of each sample were reduced to give an average spectrum and then transformed from reflectance to absorbance data. Spectra were then transformed through standard normal variate (SNV) to normalize the spectra and the second derivative was performed using the Savitzky–Golay method (Savitzky and Golay 1964) with polynomial order 2 and 11 smoothing points. For NIR classification analysis, wavelengths where no water absorption occurs were selected, as this rendered best results due to the absence of interference from different degrees of residual moisture in the samples. Wavenumbers 4000–4988, 5452–6608, and 7136–10 000 cm^−1^ were chosen as optimal regions for differentiation of strains. Principal component analysis (PCA) was performed. The classification dataset (total sample number 78) was then randomly split into a calibration and validation set at the commonly used ratio of 70/30 (calibration set 54 samples; validation set 24 samples). A linear discriminant analysis (LDA) was performed with the calibration set, using the Mahalanobis method (Mahalanobis 1936) and seven components. The created LDA was used to predict the unknown samples of the independent validation set. This was carried out in triplicate to obtain reliable results from random sample splitting.

For the culture age analysis and model extension (ii) the dataset was enlarged to include spectra of strains at different time points post inoculation (72 h, 1 w, >3 w). PCA was performed. The validity of LDA to discriminate samples by their strain at different growth stages was tested by running an LDA (Mahalanobis method; Mahalanobis 1936; eight components) on the entire sample set. A splitting into calibration/validation set was not possible as before, as not sufficient samples from different growth stages were available to be randomly included in either set.

#### HSI

For the HSI approach, images were processed using SpectrumImage R1.9.0.0030 (Perkin-Elmer Inc.) and the respective spectra for each strain were created through coaddition of appropriate image areas. Spectra were transformed from transmittance to absorbance data and SNV was performed. Water and carbon dioxide absorption regions were removed to exclude effects from moisture fluctuations in samples and atmospheric carbon dioxide on models. This approach resulted in a wavenumber region of 1000–1772 cm^−1^ that was used further on. PCA and LDA analysis were run on obtained data. For LDA, a slightly lower sample split ratio than the previously used 70/30 was employed, with 100/56 for calibration and validation set, respectively. As in the general NIR classification (i), LDA was calculated and used to predict the unknown samples of the independent validation set at three iterations.

## Results

### NIR classification (i)

**Figure 1.**
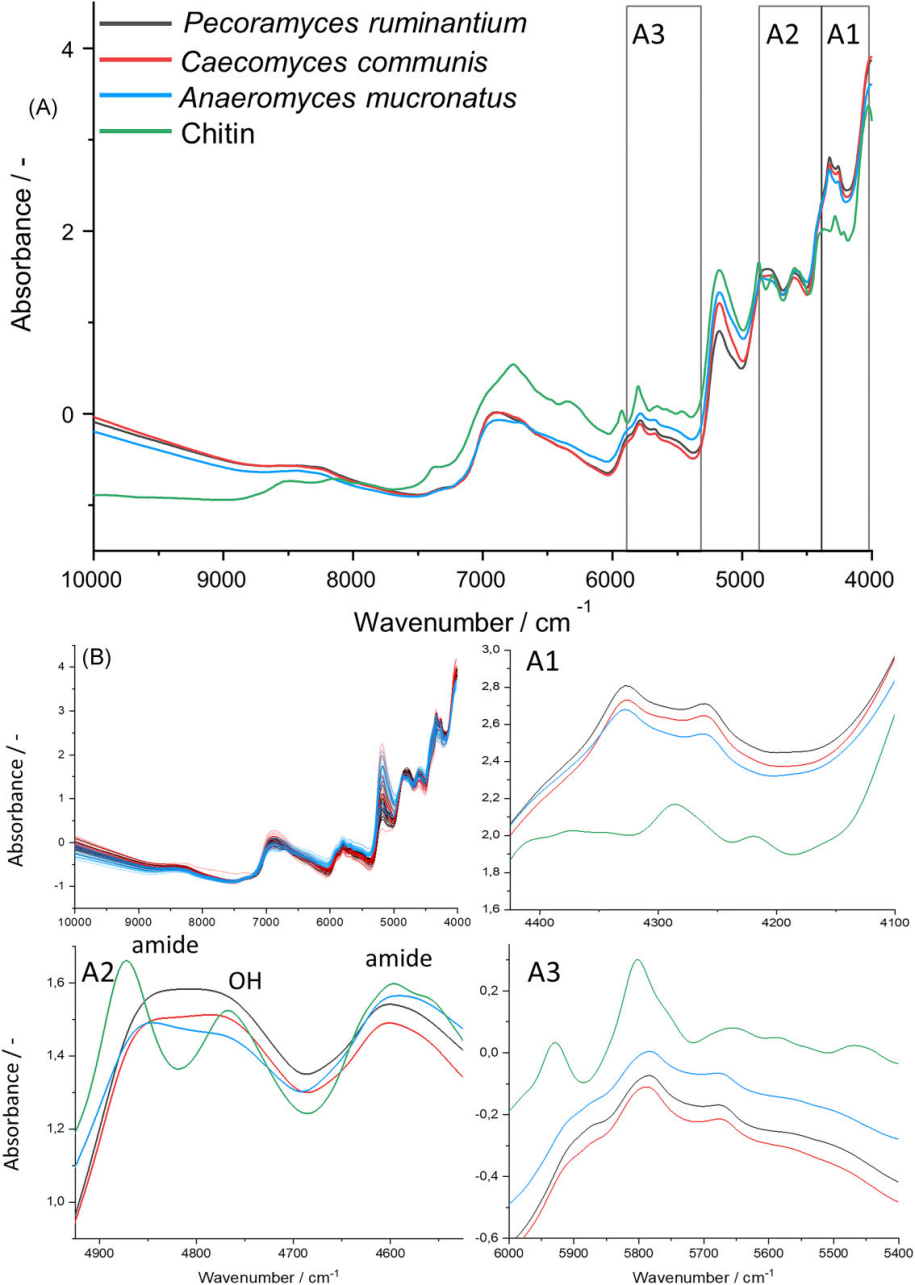
(A) Mean spectra of the respective fungal strains [*P. ruminantium* (black), *C. communis* (red), *A. mucronatus* (blue), and a spectrum of pure chitin (green)]. The spectra of the individual fungal replicates are shown with the same color coding in panel (B). Important regions, highlighted as A1-3 in Fig. 1(A) are depicted enlarged: (A1) Lipid region. (A2) Protein and carbohydrate region. (A3) C–H stretching, first overtone, and combination absorption region.

Fungal spectra showed a pattern similar to pure chitin, which is an abundant molecule in the AGF biomass (Fig. 1) (Rezaeian et al. 2004b, Gay 1991). The first observed peak (4000 cm^−1^) is a combination of C–H stretching with C–C stretching. In the highlighted region A1, AGF peaks did not match the absorption peaks of the chitin spectrum. The peaks (4324 and 4270 cm^−1^) were assigned to C–H combinations of lipids. Region A2 encompassed three distinct peaks, where the outer two (4840 and 4592 cm^−1^) are associated with amides and the central peak (4800 cm^−1^) is associated with O–H functional groups. Region A3 contained absorption from C–H combinations and C–H first overtone stretching, which however cannot be assigned to distinct biomolecules (Workman and Weyer 2007). Nevertheless, these regions were relevant for the discrimination of strains. Mean spectra of the pigmented fungal samples showed a stronger absorption at high wavenumbers (>9000 cm^−1^) when compared to the white chitin reference.

**Figure 2.**
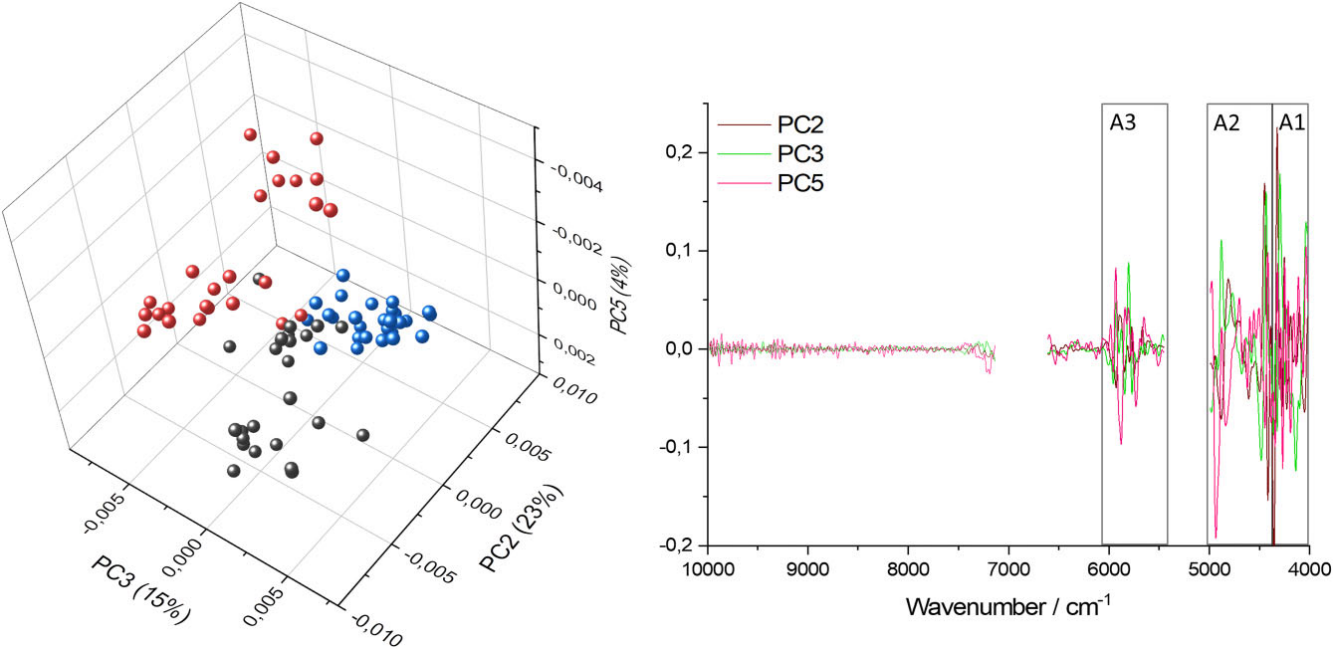
Multivariate analysis for differentiation of AGF strains by NIR. Second derivative data and absorption regions not containing water were used for multivariate analysis. On the left a PCA scores 3D plot of AGF strains [*P. ruminantium* (black), *C. communis* (red), and *A. mucronatus* (blue)] is depicted. On the right the loadings plot with the respective components is shown. The gap in the graph results from the removed water bands. Highlighted regions A1–3 are shown as in Fig. 1 (PC2: 23%, PC3: 15%, and PC5: 4%).

The three tested strains were clearly separated in PCA analysis (Fig. 2). Components 2, 3, and 5 showed the sharpest separation. While PC1 (41%) found the strongest difference among samples, it was not useful for separation of strains. PC4 (6%) only separated three *C. communis* samples and, therefore, was also not useful for strain differentiation, hence PC5 was used instead. The loadings plot emphasizes how the selected regions A1–3 (compare Fig. 1) are most important for the differentiation of strains in multivariate analysis. The used components explained 52% of the overall variance.

For the classification of strains, each random sample split resulted in a prediction accuracy of the LDA model, built on the calibration set of 100%. Each of the three independent validation sets was predicted with an accuracy of 96%.

### Culture age analysis and model extension (ii)

**Figure 3.**
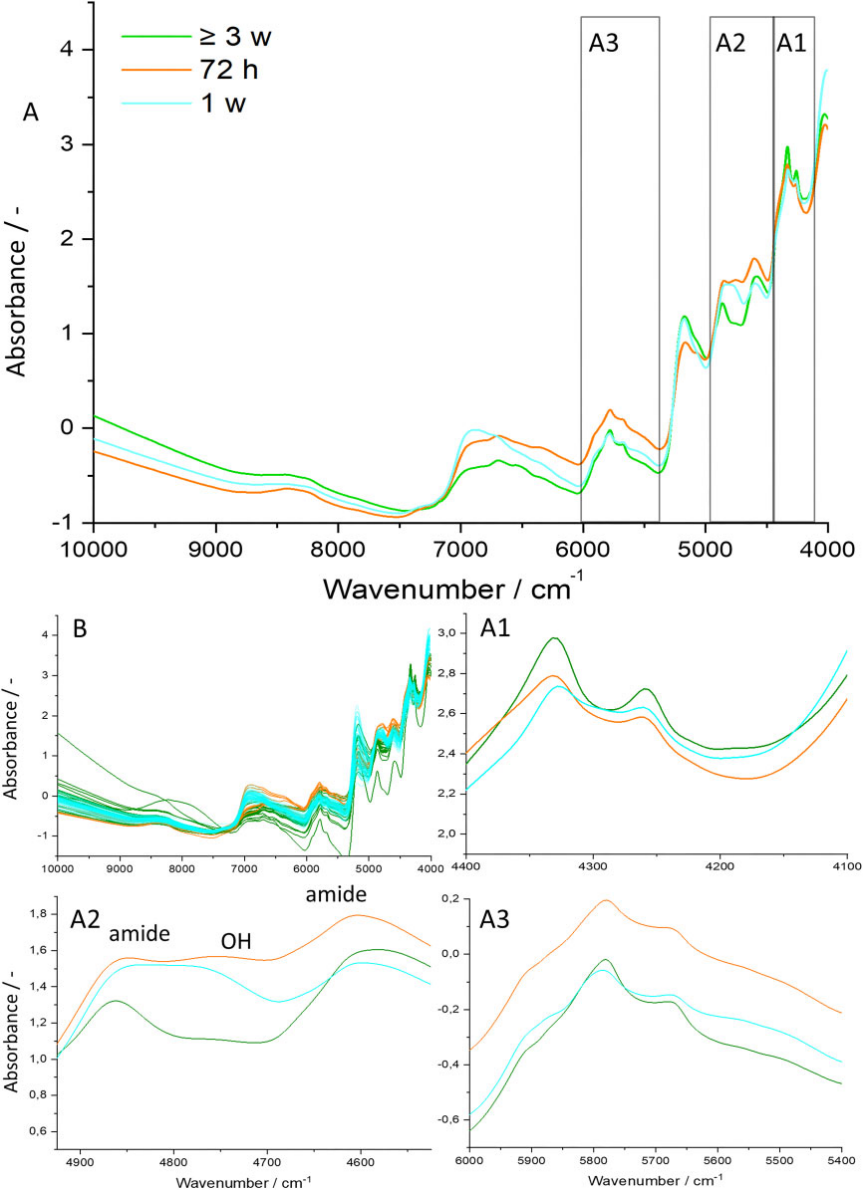
(A) Mean spectra of the respective growth time (72 h: orange, 1 w: cyan, and ≥3 w: dark green). The spectra of the individual fungal replicates are shown in panel (B) with the same color coding. Important regions are highlighted by labeled boxes (A1–3), and zoomed graphs of those regions are shown in panels A1–3 as in Fig. 1. (A1) Lipid region. (A2) Protein and carbohydrate region. (A3) C–H absorption region.

The spectra of fungal strains showed clear differences in their absorption characteristics based on culture age (Fig. 3). The oldest cultures (≥3 w) exhibited the strongest intragroup heterogeneity. In the lipid region (A1), the oldest cultures displayed the strongest absorption, while the youngest cultures did so in the C–H region (A3). In the protein and carbohaydrate region (A2), a pattern of increasing absorption of the amide bands (4840 and 4592 cm^−1^) compared to the O–H absorption (4800 cm^−1^) over time was observed. At 72 h the O–H peak almost matched the amide absorption, while in the ≥3 w samples the peak could barely be observed. The region >9000 cm^−1^ showed an absorption increase from 72 h, over 1–3-week-old cultures.

**Figure 4.**
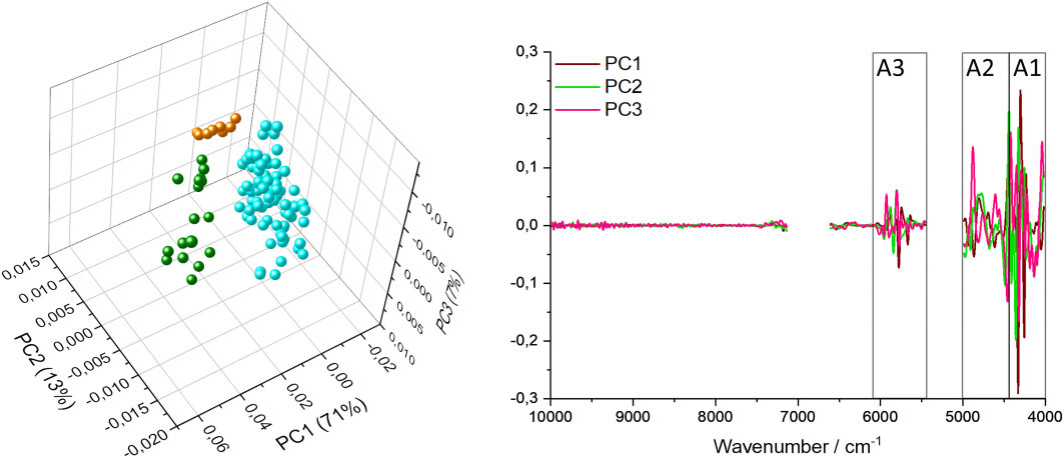
Multivariate analysis for differentiation of culture age with NIR. Second derivative data and absorption regions not containing water were used for multivariate analysis. On the left a PCA scores 3D plot of AGF strains harvested at three different time points (72 h: orange, 1 w: cyan, and ≥3 w: dark green) is depicted. On the right the loadings plot with the respective components is shown. The gap in the graph results from the removed water bands. Highlighted regions A1–3 are shown as in Fig. 3 (PC1:71%, PC2: 13%, and PC3: 7%).

The three tested culture ages were clearly separated by PCA (Fig. 4). The ≥3 w samples again showed the strongest intragroup heterogeneity. As in Fig. 2, the loadings plot emphasizes the importance of the highlighted regions (compare Figs 1 and 3) for the differentiation of different culture ages. The PCA explained 91% of the overall variance.

The prediction accuracy of the LDA model discriminating strains, calculated for the different culture ages, was 98%.

### HSI (iii)

**Figure 5.**
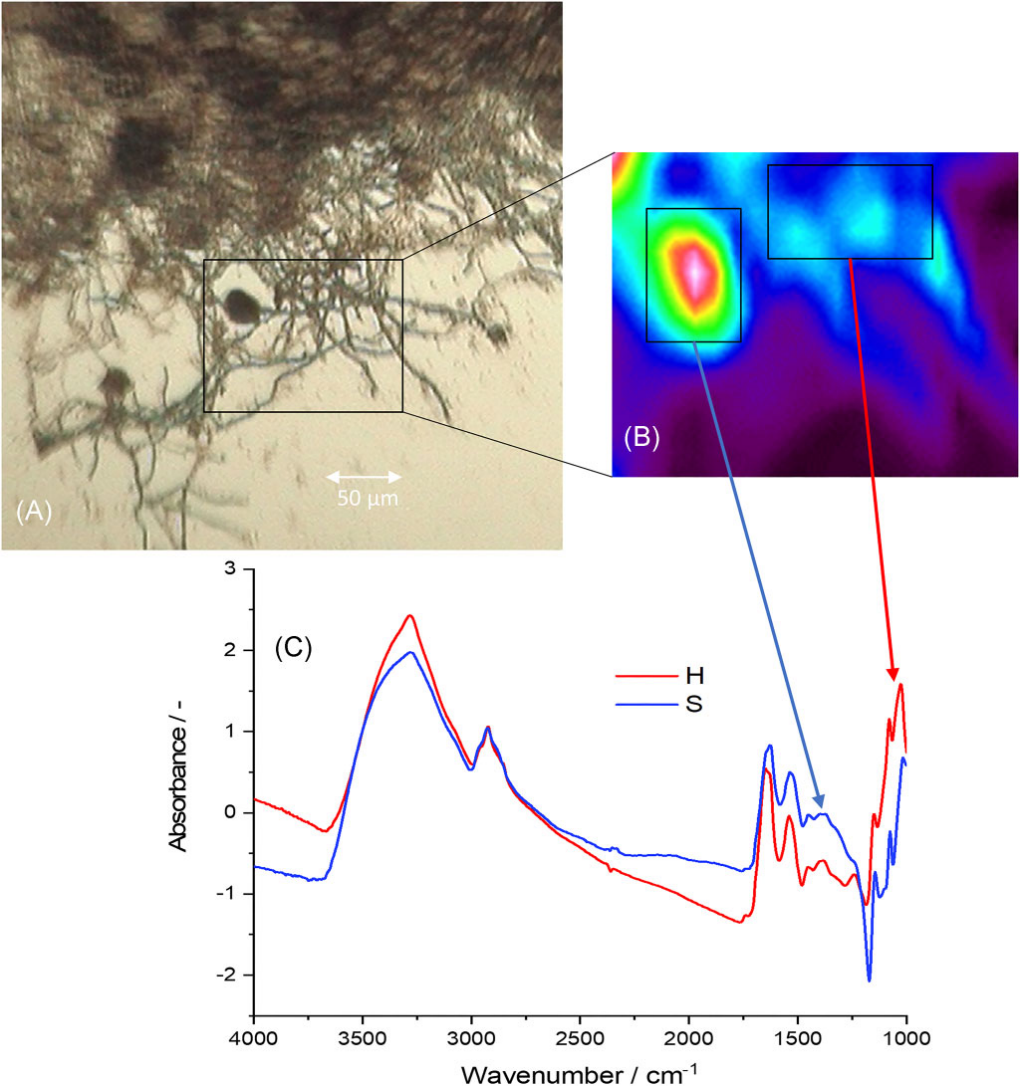
(A) Brightfield image of *P. ruminantium* with its’ respective absorption image of the highlighted region (B). (C) Coaddition of spectra within the highlighted area of sporangia and hyphae yielded the respective average spectra. This graph depicts the mean spectra of all hyphae (red) and sporangia (blue).

The general schematic procedure for generating the respective spectra with the HSI approach is portrayed in Fig. 5. HSI helped to not only gain representative spectra for each strain, but also spectra of morphologically different structures, i.e. hyphae versus sporangia. The mean spectra showed differences in their absorption characteristics in the MIR region, with the fingerprint region (1772–1000 cm^−1^) exhibiting strongest differences. The morphological structures were discriminated by PCA using the fingerprint region (appendix Supplementary Fig. 8). As the sporangial spectra showed a much greater intragroup heterogeneity, only hyphal images and images containing hyphae as well as sporangia were used for discrimination through LDA.

A strong absorption peak from O–H stretching was seen in all averaged spectra (3280 cm^−1^). As this could not only arise from chitin and carbohydrates, but also from residual moisture in the samples, the entire region was excluded for strain differentiation. This included also the C–H stretching peaks from CH_2_ (2960 and 2872 cm^−1^) and CH_3_ (2928 and 2852 cm^−1^) groups, as well as CO_2_ absorption from atmospheric carbon dioxide (ca. 2349 cm^−1^). This left the fingerprint region for robust classification to 1800–1000 cm^−1^. Here, peaks of amide I (1644 cm^−1^), arising from C=O stretching, amide II (1544 cm^−1^), arising from C–N stretching and N–H in plane bending; and amide III (1244 cm^−1^), originating from a complex mixture of functional groups present in amides, as well as peaks from chitin and proteins, were identified (López-Lorente and Mizaikoff 2016). C–H bending was also observed (1452 and 1384 cm^−1^). C–O stretching peaks associated with ethers (1152 cm^−1^), and alcohols (1080 and 1030 cm^−1^) from chitin and carbohydrates were observed. Peaks with contributions from antisymmetric (1240 cm^−1^) and symmetric (1080 cm^−1^) stretching of phosphate groups were identified, which can be associated with DNA, RNA, and phospholipids. The peak at 1240 cm^1^ was not observed in the chitin spectrum, corroborating the assignment to phosphate groups.

The three strains were clearly separated by PCA (Fig. 7). The influence of the respective peaks in the fingerprint region on discrimination can be observed in the loadings plot. The PCA explained 96% of the overall variance.

For classification, the random sample splitting resulted in an average prediction accuracy of 99% (SD ± 1%) with the LDA model. For the independent validation set an average prediction accuray of 97% (SD ± 3%) was achieved.

## Discussion

### NIR classification

NIR has been proven a suitable method for characterization of AGF biomass composition and in addition a valid discrimination method for the tested AGF strains. The general absorption trend of fungal cultures followed the absorption characteristics of chitin, which together with molecularly similar glucans, are the most abundant cell wall components of fungi (Ruiz-Herrera and Ortiz-Castellanos 2019). The differentiation of our fungal strains by the NIRS model was, however, mostly based on differences in the relative abundance of other macromolecules found in fungal cells, namely carbohydrates, lipids, and proteins. For example, the observed C–H combination peaks (Fig. 1, highlighted region A1) of the AGF samples did not match the chitin spectrum and could be a result of characteristically strong absorption of fungal lipids and their many CH_2_ groups per molecule (Hourant et al. 2000, Vieira et al. 2021). Calderón et al. (2009) found characteristic peaks from fatty acids in the NIR-spectra of Ascomycota strains at these positions, corroborating the assignment of these specific peaks to fungal lipids. Different compositions of membrane and storage lipids of AGF strains led to differences in the spectral fingerprint of the samples in region A1, allowing for differentiation of strains with our NIRS model. Chitin and other biomolecules could, however, still have an influence on this region. In the second highlighted region A2 (Fig. 1), the two observed amide peaks likely arose from protein- and chitin absorption, while the observed O–H peak likely stemmed from carbohdydrate- and chitin absorption (Workman and Weyer 2007, Ishigaki et al. 2021). They can therefore be used to estimate the relative abundance of said biomolecules in fungal biomass. *Anaeromyces mucronatus* displayed the highest amide absorption, leading to the conclusion that this strain has the highest protein + chitin to carbohydrates + chitin ratio. *Caecomyces communis* displayed the strongest O–H absorption compared to the other peaks, suggesting a lower protein + chitin content of this strain, with a relatively higher carbohydrate + chitin content. *Pecoramyces ruminantium* also exhibited high O–H absorption. Differences in the composition of fungal biomass with higher or lower abundances of protein and carbohydrates further allowed the discrimination of strains by the NIRS model. The third highlighted region A3 (Fig. 1) contained absorption from overtones and combinations of C–H stretching. C–H bonds are present in all macromolecules constituting organic biomass, and therefore a broad absorption band from many overlapping peaks is expected. Due to this strong overlap, assignment to distinct macromolecules was not possible. However, differences in relative abundance of certain molecules and hence differences in shapes of the absorption band can still be picked up by the model for correct differentiation of strains (Burns and Ciurczak 2008, Pasquini 2018).

Despite the importance of the highlighted regions, the remaining absorption regions were relevant for fungal classification as well. Models using only the wavelengths in regions A1–3 performed worse compared to models using the entire spectrum without water bands (data not shown). An explanation for this could be differences in the absorption region of C–H second overtones and combinations (roughly 8250 cm^−1^), differences in absorption of pigments near the visible light region (above 9000 cm^−1^) or influence of the C–H + C–C combination (4000 cm^−1^) (Calderón et al. 2009, Workman and Weyer 2007).

The classification of AGF strains through NIRS revealed reliable and accurate results, with a high prediction accuracy of 96% for unknown AGF samples in the independent validation set. In comparison, Schoch et al. (2012) performed a round-robin test for identification of Fungi by standard DNA barcode regions such as ITS and LSU. For early diverging lineages, which contain the Neocallimastigomycota, probabilities of correct identification of 62% for ITS and 75% for LSU were reported. One, however, has to consider that in this study discrimination of AGF strains has been carried out on the genus level with merely three out of the 22 described AGF genera. This limitation was mainly caused by the limited availability of pure cultures and with the three used being the only available pure cultures with our research consortium. Further, recently the development of various primer pairs specifically designed for detection and/or quantification of AGF strains (Kittelmann et al. 2012, Edwards et al. 2017, Young et al. 2022), as compared to standard fungal barcoding primers used by Schoch et al. (2012), have enhanced detection, identification, and discrimination of AGF. No current study, however, has investigated the success rates of AGF identification with these state of the art molecular techniques in general laboratory settings. While the comparison of identification accuracies highlights the potential of the NIRS method in combination with LDA modeling, further comparative studies are required. The corroboration of the NIRS to discriminate more strains at genus or even species level needs to be tested by including additional strains.

Besides identification accuracy, other aspects of standard nucleic acid-based techniques should be considered. Schoch et al. (2012), for example, reported a PCR amplification success rate of only 65% for early diverging fungal lineages. As the NIRS method does not require PCR amplification this is an additional beneficial feature of this approach for AGF assignment to reference spectra. Further, the proposed method does not require chemicals for nucleic acid extraction and sequencing (Rittenour et al. 2012), making it preferable considering the principles of green chemistry (DeVierno Kreuder et al. 2017), and eliminating potential biases brought in by nucleic acid extraction and PCR.

An additional benefit of NIRS is the nondestructive nature of the technique that allows for further downstream analysis of samples. In this study, further measurements were conducted with the same samples to differentiate strains through DART mass spectrometry and MALDI-TOF MS (unpublished results).

To the best of our knowledge, this is the first study to report the use of NIR for the identification of fungal biomass. As could be shown for the selected AGF, this method allows the simple, fast, and nondestructive distinction of fungal pure cultures. With the develop model, the three selected cultures can be discriminated and unknown cultures from these strains identified. The inclusion of more known strains as pure culture references could enable the development of a model for discriminating many more AGF strains. It could therefore be used as an alternative approach to molecular identification and characterization of strains, a common practice in standard laboratory settings with MALDI-TOF MS (Hendrickx 2017, Gómez-Velásquez et al. 2021). It bears the additional benefit of being cheaper and requiring less chemicals than MALDI-TOF MS. However, NIRS identification requires fungal samples to be acquired in pure culture and with sufficient biomass to carry out measurements. The HSI approach circumvents these limitations by adding spatial resolution to the data that allows for characterization on a small scale, with little biomass and the ability to separate fungi from particular plant matter found in growth media, or using mixed cultures for analyses (see HSI).

### Culture age analysis and model extension

As the growth and maturation of fungal strains corresponds to the formation of different quantities of certain macromolecules, AGF biomass can exhibit different amounts of chitin, protein, carbohydrates, or lipids depending on the culture age or growth phase (Phillips and Gordon 1989, Gay 1991). Since differentiation of strains in the NIRS model is based on the relative abundances of these macromolecules, culture age could have a significant effect on the robustness of the model. We therefore investigated whether identification of strains was still possible with strains harvested after different growth times. We tested cultures grown for 72 h, 1 week, and over 3 weeks, to reflect cultures in their most active growth phase, mature-, and eventually decaying cultures, respectively.

The LDA of AGF samples from different culture ages rendered a good prediction accuracy of the model for discriminating the strain (98%). With the limited number of samples from each strain at different culture ages, a random splitting of the sample set into calibration and validation set was not possible. Despite the shortfall of external validation, the accurate model predictions showed that discrimination of samples by strain was still possible when different culture ages are used. As only one additional component was employed compared to the LDA model used for aim (i), the chance of overfitting was low (Reyna et al. 2017). With a larger sample set, including more strains harvested at each selected time point, a more robust model, encompassing various different culture ages could therefore be designed and verified by external validation (Reyna et al. 2017, Pasquini 2018).

Overall, 3-week-old samples showed the largest intragroup heterogeneity (Figs 3B and 4). This is likely a result of the largest time interval in between individual samples of this group, with up to 2 months. Nevertheless, taking this into account, the >3 w samples still clustered well together in multivariate analysis.

We further investigated the changes in biomass composition over time through interpretation of NIR absorbance of the respective culture ages and found clear differences in the characteristics of the mean spectra (Fig. 3). Especially in region A2, a clear shift of absorption over time was observed. Here, the intensities of the O–H band decreased, while the absorption of the amide bands increased, when moving from younger to older cultures. This indicates a decrease of carbohydrate + chitin and an increase of protein + chitin ratios in the fungal biomass over time. This result was also corroborated by previous studies. Gay (1991), for example, used biochemical methods to determine the protein and chitin content of AGF strains and found an increase in protein and more significantly an increase in the protein to chitin ratio over time. Phillips and Gordon (1989) used biochemical methods to determine the storage carbohydrate content of AGF strains and found the highest amounts of carbohydrates in fungal biomass during active growth from 18 to 75 h. Interpretation of the absorption of C–H functional groups in regions A1 and A3 was not as straightforward, as changes over time could result from a multitude of different biomolecules, as described before (see NIR classification).

The increase of absorption with culture age in the region >9000 cm^−1^ could be caused by an increase of pigment content, which absorbs in the visible light region and absorption shoulders reaching into the NIR range at high wavenumbers (Calderón et al. 2009, Muniz-Miranda et al. 2019). Dark coloration of fungal cultures over time is commonly observed in AGF cultures and is believed to be some kind of a stress response and may even correlate to the formation of pigmented aero-tolerant resting stages (Wubah et al. 1991). While the absorption in this region matched pigmentation patterns, it could also be stemming from commonly observed scattering effects at high wavenumbers in the NIR region. These artifacts could lead to apparent differences in spectra, due to different particle sizes of the dried fungal biomass matrices (Burns and Ciurczak 2008, Xie and Guo 2020). Interestingly, despite clear differences in the absorption in this region, it had very little influence on the separation of samples for the LDA based on culture age (see loadings plot; Fig. 4).

In addition to culture age, the selected medium for fungal growth could have significant influence on the observed spectral patterns of each strain. A switch from cellobiose and xylan as used in these experiments to other C-sources could lead to different relative abundances of macromolecules in AGF biomass or changes in the speed of accumulation of macromolecules over time. For instance, when cultures in our experiment were grown on the same medium with the addition of yeast extract, a much faster build up of proteins was observed (data not shown). Due to these influences, a standardized medium as well as growing time is recommended when using NIR as a characterization and discrimination tool for AGF. Additional studies could shed light on the influences of media composition on AGF biomass build up over time.

While a proof of principle for identifying AGF strains from different culture ages has been shown with this study, further investigations with larger sample sets are needed to robustly confirm the applicability of the NIRS method to classify AGF strains at different culture ages. The additional use of HSI could help to shed light on the influence of certain morphological structures on the overall biochemical composition of fungal biomass.

### HSI

The HSI approach yielded accurate and reliable classification results, with a prediction accuracy of 97% in independent validation sets. This was even higher than the classification accuracy of the NIRS model (see NIR classification), corroborating HSI as another sound alternative for AGF strain classification. With HSI, only minimal biomass is needed and a few hyphae can generate sufficient absorption data for spectroscopic classification of strains. This not only enables specific detection and differentiation of strains not available in pure culture, it also overcomes one of the main limitations of the NIRS approach, which requires larger amount of biomass. An additional benefit is the ability to harvest consecutively limited amounts of biomass from the same culture bottle, facilitating the study of fungal biomass over time, as compared to the necessity of whole culture harvest for the NIRS method.

**Figure 6.**
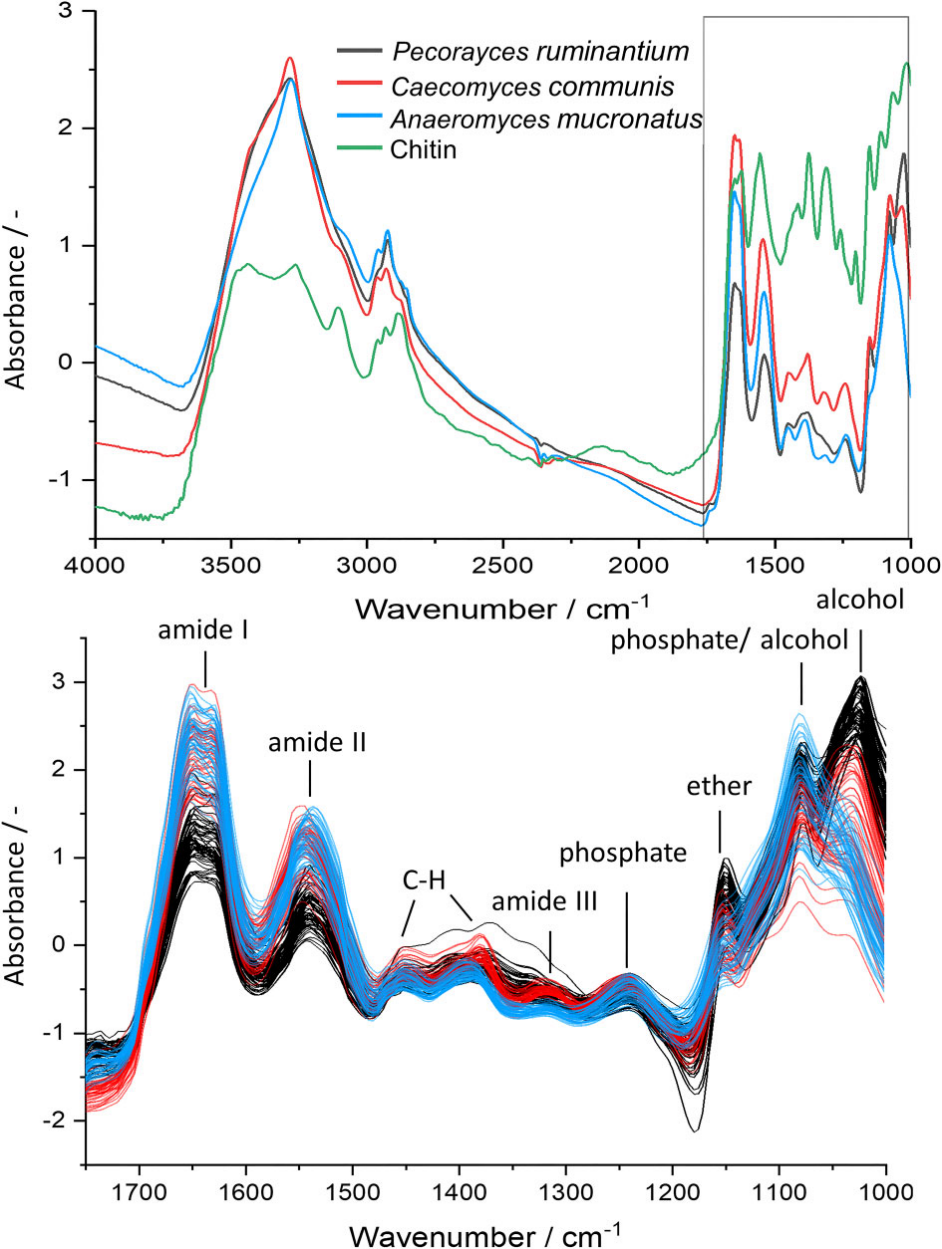
Top: mean HSI-spectra of fungal strains [*P. ruminantium* (black), *C. communis* (red), and *A. mucronatus* (blue)] and chitin reference (green). The fingerprint region is highlighted as it is most relevant for differentiation of samples. Bottom: Individual spectra of AGF samples in the zoomed-in fingerprint region are shown with the same color coding as above including peak assignment to functional groups.

Due to the utilization of MIR compared to NIR, the assignment of peaks in HSI spectra was more straightforward. With MIR, fundamental excitations can be correlated to distinct functional groups, compared to the difficult interpretation of combination bands in NIR. An example is the direct assignment of peaks to phosphate groups (e.g. 1240 cm^−1^; see Fig. 6). Absorption from this functional group cannot be detected with NIR, as overtones, which occur at roughly double the wavenumber as the fundamental excitation, would only be detectable in the MIR region. However, these overtones are not detected in MIR, as they exhibit much lower absorption than fundamental excitations, leading to them being concealed. Combination bands of C–H stretching with phosphate groups could occur in the C–H region of the NIR spectrum (Fig. 2, region C), as the sum of the individual wavenumbers (ca. 2900 and 1240 cm^−1^, respectively) adds up to just over 4000 cm^−1^. However, these bands also contain more frequently occurring C–H stretching plus C–H bending combinations (ca. 2900 and ca. 1400 cm^−1^), masking phosphate contribution (Burns and Ciurczak 2008, Pasquini 2018, Beć et al. 2022).

**Figure 7.**
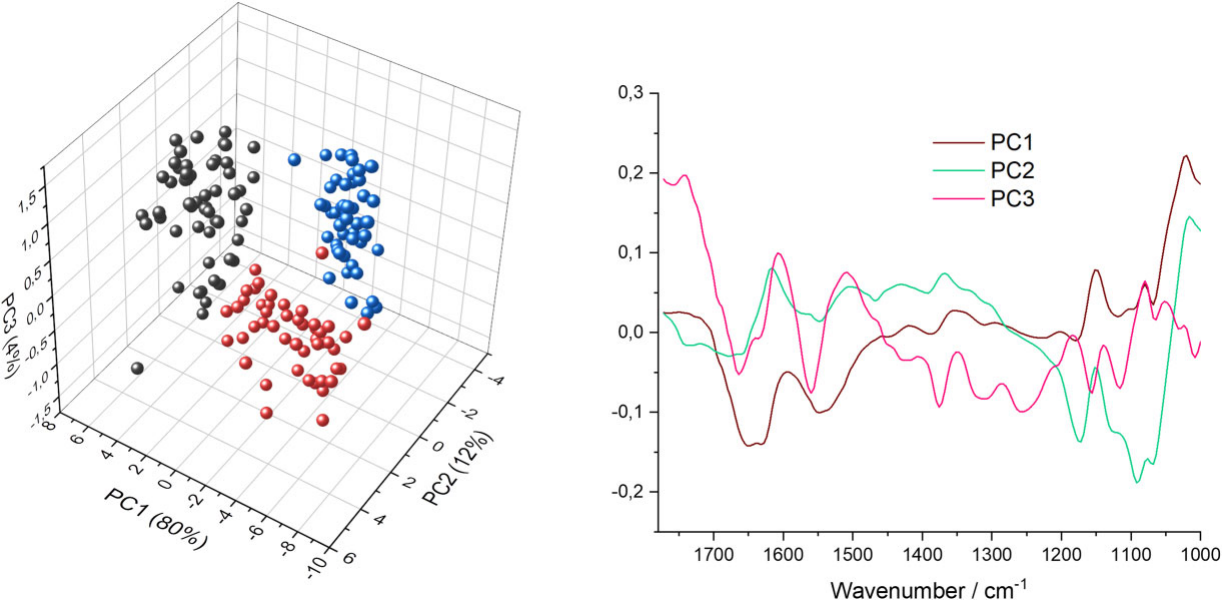
Multivariate analysis for differentiation of AGF strains with the HSI approach. On the left a PCA scores 3D plot of AGF strains [*P. ruminantium* (black), *C. communis* (red), and *A. mucronatus* (blue)] is depicted. On the right the loadings plot shows the influence of the fingerprint region (1772–1000 cm^−1^) on the discrimination of the respective strains. The PCA ordination explains for 96% of toal variance (PC1: 80%, PC2: 12%, and PC3: 4%).

Overall, individual fungal spectra showed clear differences in their spectral fingerprint between strains, while strong similarities were observed for samples from the same strain. The most prominent peaks in the MIR spectra of AGF strains were the amide peaks (see Fig. 6) that are associated with chitin as well as with protein. Furthermore, ether or alcohol functional groups contributed to the C–O stretching that was associated with carbohydrates or chitin and observed in all AGF strains (Salman et al. 2010, Prabu and Natarajan 2012). The same region of the spectra, however, might also be influenced by phospholipids, DNA, and RNA absorption (Parker and Quinn 2013). Interestingly, this region was very prominent for *A. mucronatus* samples, possibly overshadowing absorption at other wavelengths. This could be a result of strong absorption from membrane lipids, RNA, or DNA molecules for this strain. *Anaeromyces mucronatus* displays polycentric, filamentous growth, with many highly branched and constricted hyphae (Breton et al. 1990). This overlap of multiple hyphae could result in strong absorption from phospholipids in this region. A contribution from more evenly distributed nuclei and associated DNA could also be an explanation for this strong absorption. Overall, the absorption from this region had a strong influence on the discrimination of strains in multivariate analysis (Fig. 7).

PCA of morphological structures of *P. ruminantium* specimen revealed a clear separation of hyphae and sporangia (appendix Supplementary Fig. 8) with sporangia displaying higher heterogeneity than hyphae. This stronger heterogeneity of sporangia spectra can be explained through different degrees of development of sporangia. Young sporangia could exhibit different composition of macromolecules compared to fully mature sporangia. Furthermore, hyphae were scanned as networks with multiple overlaying structures, hence already representing a complex image.

HSI allows for differentiation of morphological structures for *P. ruminantium* samples and reveals differences in their composition due to the respective absorption intensities. *Anaeromyces mucronatus* was not investigated in this way, as the strain available in our lab sterilely reproduces through hyphal growth and therefore does not contain sporangia, leading to morphologically homogeneous thalli (Fliegerová et al. 2002). The bulbous thalli of *C. communis* with little to no hyphal structures (e.g. holdfasts), show little morphological variation (especially in dried samples), which is why this strain was not investigated. The approach for detecting morphological differences in AGF cultures could, however, easily be extended to other monocentric or nonsterile polycentric AGF strains.

Overall, HSI gives us the advantage of visual and spectral information on the same sample (Wang et al. 2018, Soni et al. 2022, Wu et al. 2022). It could therefore be used to address similar research questions as FISH, while circumventing the need to design specific probes and protocols (Moter and Göbel 2000, Baschien et al. 2001). Future research could focus on the identification of strains growing in coculture, as individual AGF could be visually distinguished from one another and then classified by their respective spectral pattern. As such an experiment with competitive exclusion in artificial cocultures could be considered. Furthermore, AGF are often cultivated in media containing plant material (e.g. wheat straw, rice straw, and sorghum; Stabel et al. 2021, Vinzelj et al. 2022), which makes classification with NIR more challenging, as the plant material remains in the sample after processing. With spatial resolution, the absorption from plant particles could be avoided, and through prior collection of reference spectra from plant material and fungi, HSI could also allow for the visual differentiation of the two by the respective spectral information (Lu et al. 2020, Soni et al. 2022). With the present study, we could, however show a fundamental proof of concept.

In general, the HSI approach could potentially lead to classification of AGF from environmental samples, such as animal feces or rumen. Rezaeian et al. (2004a) described the visual detection of AGF from digested plant material taken from sheep intestines. As such, rumen content or feces could be microscopically scanned for fungal thalli, of which representative spectra could be recorded. The spectral information could then be used to assign found fungal thalli to specific species. This could give information about the presence and frequency of occurrence of specific species in environmental samples, and hence enable a culture-independent method for AGF classification. However, prior formation of novel models including many, even yet uncultivated, AGF strains and assessment of the matrix effects of different intestines and feces contents on AGF classification would be required for reliable applications.

## Conclusion and outlook

The described NIRS method demonstrates a simple and accurate approach for AGF classification. As a proof of principle, we could also demonstrate that using cultures of different age still enabled separation of strains by a discriminant model. However, pure cultures with sufficient biomass are required for the NIRS method.

The HSI approach only requires minimal biomass and adds spatial resolution to the gathered data. This enables the classification of strains with little material available and could even allow for classification in medium containing particular matter or other impurities. Future studies could look into the possibilities of AGF classification harvested from more complex growth substrates, which could potentially even enable AGF classification from environmental samples, leading to a culture-independent classification approach. For this, hurdles, such as matrix effects of different environmental samples and the inclusion of more strains, even uncultured ones, would need to be overcome.

In the present study, three strains were classified on the genus level. With the availability of more pure AGF strains, the current approaches could be tested on a larger set of genera and the ability to differentiate strains even at the species level could be evaluated. This is especially interesting for strains with monocentric, filamentous growth, which currently cannot be readily distinguished by light microscopy. The inclusion of a larger set of AGF strains would also be essential to achieve reliable results for classification in environmental samples. We thus also advocate for the inclusion of spectroscopic (NIR/MIR) characterization of novel strains, to generate a comprehensive database of so far isolated strains.

## Supplementary Material

xtae025_Supplemental_Files
