# Supplementary material for: Exploring near-infrared spectroscopy and hyperspectral imaging as novel characterization methods for anaerobic gut fungi
Source: FEMS Microbes. 2024 Sep 10;5:xtae025. doi: 10.1093/femsmc/xtae025 (PMC11412074; doi:10.1093/femsmc/xtae025)
Supplement: xtae025_Supplemental_Files [file xtae025_supplemental_files.zip › FEMSMC-2024-016.R1 one sentence summary.docx]

Near-infrared spectroscopy and hyper-spectral imaging are explored as novel methods for characterization of anaerobic gut fungi strains, independent of molecular approaches.
