# Supplementary material for: Exploring near-infrared spectroscopy and hyperspectral imaging as novel characterization methods for anaerobic gut fungi
Source: FEMS Microbes. 2024 Sep 10;5:xtae025. doi: 10.1093/femsmc/xtae025 (PMC11412074; doi:10.1093/femsmc/xtae025)
Supplement: xtae025_Supplemental_Files [file xtae025_supplemental_files.zip › supplementary_material_NIRAGF_revised.docx]

# **Supplementary Material**


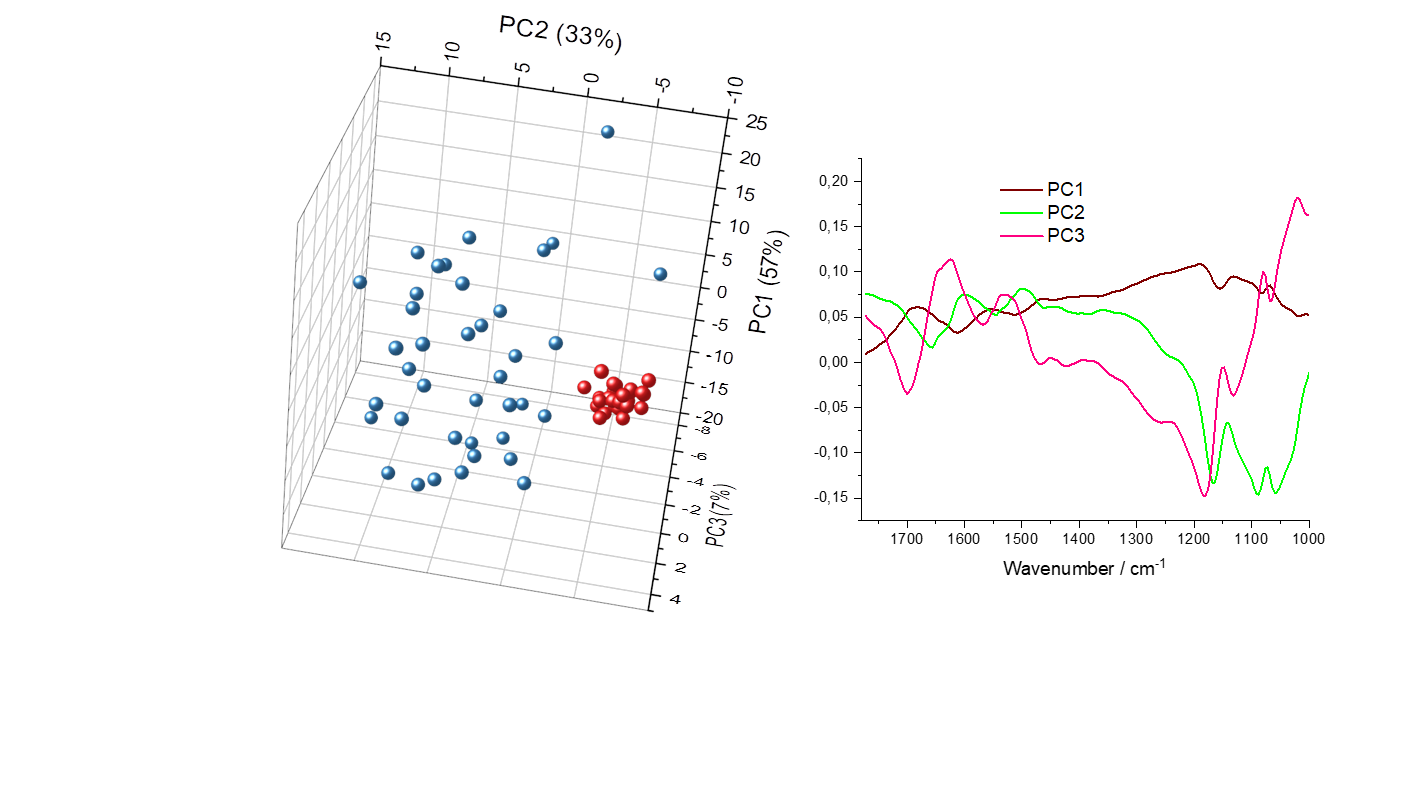


Figure 8: Multivariate analysis for discrimination of morphological structures of *Pecoramyces ruminantium*. SNV data of the fingerprint region (1 772 – 1 000 cm^-1^) was used. On the left PCA scores 3D plot of sporangia (blue) and hyphae (red) are shown. The loadings plot shows the influence of the fingerprint region on the discrimination of the morphological structures (right). (PC1: 57%, PC2: 33%, PC3: 7%)


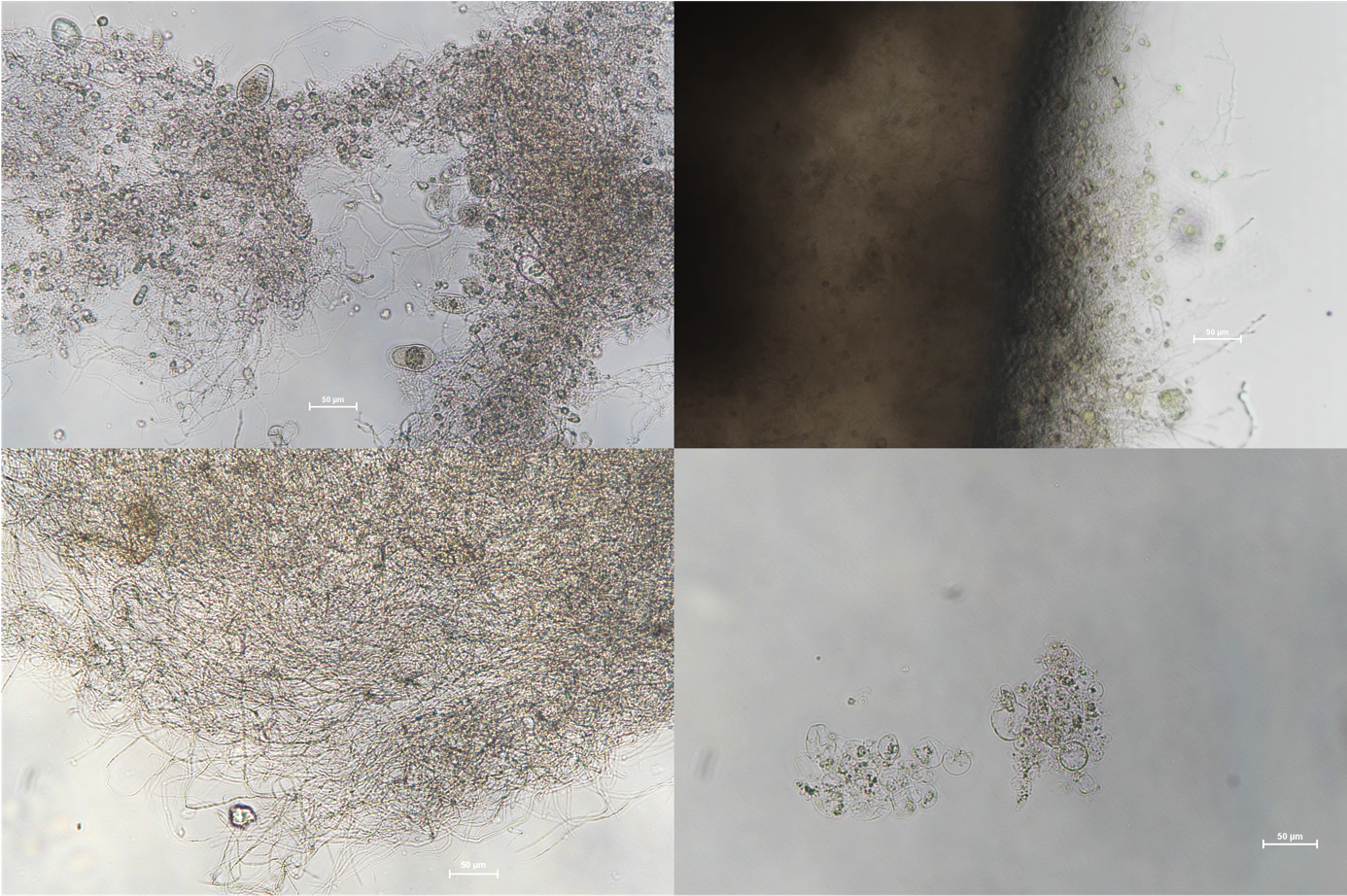


C

D

B

A

Figure 9: Microscopy images of the used AGF strains. A) *Pecoramyces ruminantium* showing distinct morphological structures with hyphae and sporangia present. B) Aggregate of *Pecoramyces ruminantium*. Aggregation of cultures was often observed after longer incubation. C) *Anaeromyces mucronatus* displaying polycentric, filamentous growth. D) *Caecomyces communis* displaying monocentric, bulbous growth.


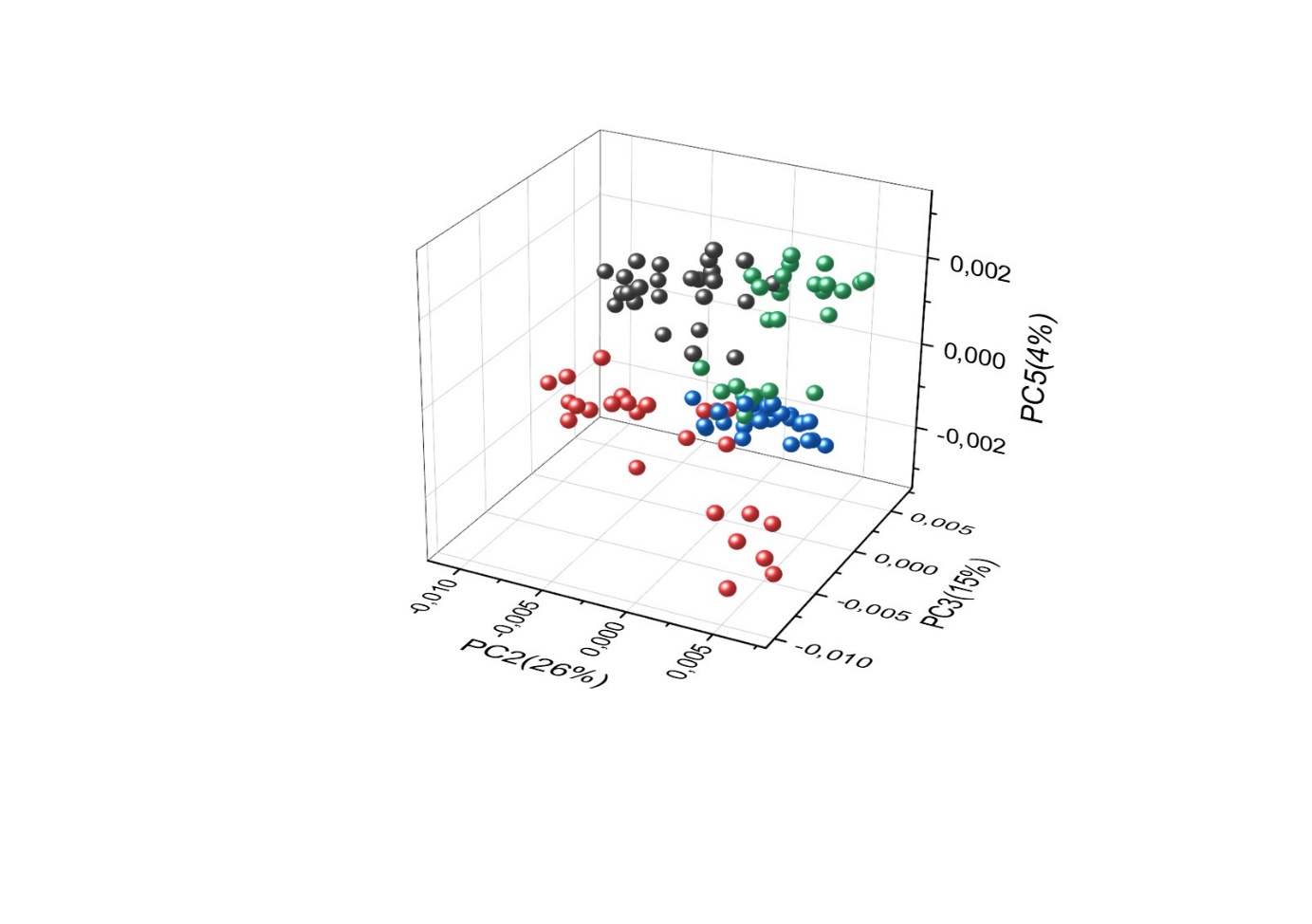


Figure 10: Multivariate analysis for differentiation of four AGF strains by NIR (*Pecoramyces ruminantium* (black), *Caecomyces communis* (red), *Anaeromyces mucronatus* (blue), *Neocallimastix frontalis* (green)). Second derivative data and absorption regions not containing water bands were used for multivariate analysis. The strain *Neocallimastix frontalis* was excluded from further analysis, as methanogenic archaea were detected in small amounts in the culture (PC2: 26%, PC3: 15%, PC5: 4%).
