# Supplementary material for: Exploring near-infrared spectroscopy and hyperspectral imaging as novel characterization methods for anaerobic gut fungi
Source: FEMS Microbes. 2024 Sep 10;5:xtae025. doi: 10.1093/femsmc/xtae025 (PMC11412074; doi:10.1093/femsmc/xtae025)
Supplement: xtae025_Supplemental_Files [file xtae025_supplemental_files.zip › FEMSMC-2024-016.R1 supplementary data - reviews and author response.docx]

**Reviews of manuscript FEMSMC-2024-016.r1**

**Reviewer 1**

**Recommendation: Minor Revision**

The manuscript „Exploring Near-Infrared Spectroscopy and Hyperspectral Imaging as novel Characterization Methods for Anaerobic Gut Fungi" by the authors Neurater et al. is an interesting work that shows a new possible method for the identification of anaerobic gut fungi (AGF). The approach used is innovative and provides a novel sequence-independent tool for the characterization of AGF. In my opinion, the work is suitable for publication in the journal FEMS Microbes, but I would recommend some minor corrections before acceptance.

Line 32: „growth age“: this term, which appears several times in the text, is in my opinion not quite appropriately chosen and I would suggest e.g.: „cultures of different growth phase/cultivation time".

Lines 59-60: I do not think that „monocentric/polycentric growth“ is the right term, I would suggest :…species/genera with monocentric/polycentric thallus...“

The chapter in lines 54-62 should be expanded to include a brief description of genera with bulbous rhizomycelium, especially if Caecomyces was included in the experiments.

Line 66: Add percentage of AT content

Lines 85-92: I suggest rewriting this chapter to make it more understandable. …“ configuration of the microbial cell….... provides insight into the biochemical composition…“ ???

Line 141: I suggest rewriting this sentence to give a more precise description, because the cultures were not used for differentiation, but were tested as reference strains for possible differentiation by spectrophotometric methods.

Line 172: for getting fungal powder I would expect using a pestle, not a spatula ???

Chapter Sample preparation (lines 166-184): Consider whether a table with all samples would be clearer for readers (just a suggestion).

Chapter Data processing

Line 229:… measurements were reduced??? How is this to be understood?

Line 232: Savitzky- Golay: add references

Line 233: I suggest rewriting „wavelengths containing no water absorption..“, e.g. „wl at which no water absorption occurs

Line 240, 246: Mahalnobis: add references

Line 243: I suggest „for analysing the culture at different growth stages..."

Line 256: Explain abbreviation SNV

Chapter Discussion

Line 346: method for tested (or selected) AGF strains.

Concluding that the methods used are suitable for characterization of AGF would require analysis of all genera.

I am not sure if the conclusions are not too optimistic. For example, it would be interesting to compare morphologically similar species (Caecomyces x Cyllamyces). Also, with the analysis of the mixed samples (faeces), the conclusions would be more cautious,as the analysis shows changes in different growth phases and also depending on the substrate. Please, discuss it shortly.

**Reviewer 2**

**Recommendation: Minor Revision**

Neurauter et al. explored the utility of NIRS and HSI as new methods for classification of AGF. This manuscript was an interesting read. I am very interested in research on this group of fungi. While the approach could be innovative and offer faster results, the utility was not entirely clear to me. My concerns are numbered below.

1. The authors used three strains belonging to three different genera for their proof of principal. This approach is good. My concern was with the choice of strains. The three genera are representatives of the three morphological varieties of AGF: filamentous monocentric, filamentous polycentric, and bulbous. Their spectra are expected to of course differ and the PCA is also expected to differentiate them into three clusters owing to their morphological differences. What if the same approach was used with three monocentric filamentous genera. Would the spectra be variable and would the PCA analysis be able to cluster them? If this is to be used on a larger scale beyond the proof of principal study, this has to be addressed. I understand that the authors might not have access to more strains due to the lack of a culture collection for AGF. Can they maybe add one more of each morphology to their experimental design. This will help tremendously.

2. I am still not clear on how this approach would be used for classification of pure cultures. Would one need to run strains of known identity each time an unknown sample is to be classified? If that is the case, then maybe more clarification on that can be added to the paper.

3. Also, the discussion on extending the use of this beyond pure cultures into fecal or rumen samples was not clear. Would the results from such analysis be similar to a culture-independent study and the components of the sample can be reported on as % abundances? More clarification is needed for that.

Otherwise, this was interesting and I would like to see the application on a wider scale in the AGF world.

**Editor Decision: Minor Revision**

**Author's Response to Decision Letter for (FEMSMC-2024-016)**

Dear Prof. Scott!

We are pleased with the positive reception of our manuscript and that both reviewers and editor saw merit and interest in our work. Using the constructive suggestions offered by both reviewers, we have clarified certain sections of the manuscript, as highlighted in the reviewer commentaries. Please find below in blue our explications to all reviewer comments including line references to the revised manuscript. We thank the editor and reviewers for their constructive comments and feel certain that the revised version of the manuscript has substantially improved and is eligible for publication in FEMS Microbes.

Sincerely,

Markus Neurauter (on behalf of all co-authors)

Reviewer Comments to Author/s:

Reviewer: 1

Comments to the Author

The manuscript „Exploring Near-Infrared Spectroscopy and Hyperspectral Imaging as novel Characterization Methods for Anaerobic Gut Fungi" by the authors Neurauter et al. is an interesting work that shows a new possible method for the identification of anaerobic gut fungi (AGF). The approach used is innovative and provides a novel sequence-independent tool for the characterization of AGF. In my opinion, the work is suitable for publication in the journal FEMS Microbes, but I would recommend some minor corrections before acceptance.

We are pleased that the reviewer recognizes the value of our work and considers it a good fit for the scope of the journal.

Line 32: „growth age“: this term, which appears several times in the text, is in my opinion not quite appropriately chosen and I would suggest e.g.: „cultures of different growth phase/cultivation time".

We agree with the reviewer that the term “growth age” is not the best choice. The term in the abstract has in fact been overlooked in the correction of the manuscript and has been changed to “culture age” throughout the rest of the text, which we deem more appropriate. We initially decided against the term “growth phase”, as the inoculum taken from other AGF cultures was not in a distinct growth phase (e.g. as when harvesting only zoospores) and therefore we could not ensure a distinct “growth phase” at a certain time after start of cultivation. The term has now been clarified in the Abstract (Line 32) and “culture age” is used throughout the manuscript.

Lines 59-60: I do not think that „monocentric/polycentric growth“ is the right term, I would suggest :…species/genera with monocentric/polycentric thallus...“

This is a good point and has been revised as suggested (Lines 59-61)

The chapter in lines 54-62 should be expanded to include a brief description of genera with bulbous rhizomycelium, especially if Caecomyces was included in the experiments.

A brief description of bulbous thallus development has been added in Line 62.

Line 66: Add percentage of AT content

Good suggestion, the information has been added (Line 68).

Lines 85-92: I suggest rewriting this chapter to make it more understandable. …“ configuration of the microbial cell….... provides insight into the biochemical composition…“ ???

We have rewritten the paragraph and hope it is clearer now (Lines 89-96)

Line 141: I suggest rewriting this sentence to give a more precise description, because the cultures were not used for differentiation, but were tested as reference strains for possible differentiation by spectrophotometric methods.

The reviewer is correct that strains were used as reference strains for possible differentiation, however, the prediction of unknown samples was carried out on the same three strains. The strains were therefore used as reference strains as well as for differentiation and we believe that describing them as reference strains might be misleading (now Line 145).

Line 172: for getting fungal powder I would expect using a pestle, not a spatula ???

Thank you for pointing this out! The used description is misleading. The lyophilized fungal powder was in fact broken apart in the Epi with a spatula and then transferred to the tubes. The powder is already very fine and does not need grinding. We have changed it to “fungal powder was homogenized with a spatula” in Line 176 to avoid confusion.

Chapter Sample preparation (lines 166-184): Consider whether a table with all samples would be clearer for readers (just a suggestion).

We thank the reviewer for this constructive comment, but due to space limitations and the already very concise but informative content of chapter 2.2. (sample preparation) we have refrained from adding another table to the manuscript.

Chapter Data processing

Line 229:… measurements were reduced??? How is this to be understood?

This means an average was calculated by creating one mean spectrum of the three technical replicates. The term reduced is commonly used in pre-processing of spectra and was hence used in the methods section. The sentence was changed to “to give one average spectrum” to be more clear (Line 233).

Line 232: Savitzky- Golay: add references

The reference has been added (Line 236)

Line 233: I suggest rewriting „wavelengths containing no water absorption..“, e.g. „wl at which no water absorption occurs

The sentence has been changed as suggested (Line 238).

Line 240, 246: Mahalnobis: add references

The reference has been added in Lines 245 and 251.

Line 243: I suggest „for analysing the culture at different growth stages..."

As previously described, we deem the term “growth stage” not appropriate, as not all parts of the culture can be ensured to be in the same growth stage as would be the case when using inoculation with only zoospores. We chose this wording here as it fits the title of aim (ii) and we believe it is clearer for readers to follow the same structures of aims in the different sections of the manuscript.

Line 256: Explain abbreviation SNV

The abbreviation and the aims of the technique are now described in Line 235.

Chapter Discussion

Line 346: method for tested (or selected) AGF strains.

Concluding that the methods used are suitable for characterization of AGF would require analysis of all genera.

The reviewer is right, the sentence has been changed for clarification as suggested (Line 351).

I am not sure if the conclusions are not too optimistic. For example, it would be interesting to compare morphologically similar species (Caecomyces x Cyllamyces). Also, with the analysis of the mixed samples (faeces), the conclusions would be more cautious, as the analysis shows changes in different growth phases and also depending on the substrate. Please, discuss it shortly.

We agree with the reviewer that the comparison of morphologically similar species would be a very interesting approach with this technique. However, the AGF availability in pure culture is very restricted and in this study we used all strains (i.e. in pure culture, without frequently co-occuring methanogens) currently available in our lab. Initially, Neocallimastix frontalis was included in the analysis to compare two species of monocentric, filamentous fungi and results looked very promising for separation of it with Pecoramyces ruminantium. However, we discovered that methanogenic archaea were occurring in small amounts in our Neocallimastix frontalis cultures during the course of our experiments. We excluded this strain to ensure that the differentiation of strains is solely based on differences in strain characteristics and not the presence/absence of methanogens. We have now included the initial testing of this strain in the discussion (Lines 419-426), as well as an additional supplementary figure, which displays the separation of the four strains, with two morphologically similar strains, in PCA analysis.

We also agree that the analysis of mixed samples and feces would be challenging due to the different growth phases present and the matrix effects of the substrate. We have discussed the influences of growth phase and that our results suggest they could be overcome with models based on larger datasets (Discussion lines 452-461 and 502-506). The hurdles with matrix effects of environmental samples were added in Discussion lines 583-588.

We have added more cautionary statements to the Conclusion section (Line 601-603) and retitled it as “Conclusion and Outlook” to emphasize that future possibilities and not current applications are discussed.

Reviewer: 2

Comments to the Author

Neurauter et al. explored the utility of NIRS and HSI as new methods for classification of AGF. This manuscript was an interesting read. I am very interested in research on this group of fungi. While the approach could be innovative and offer faster results, the utility was not entirely clear to me. My concerns are numbered below.

We are pleased to hear that the reviewer was interested in our approach and appreciates the advantages the techniques have to offer for AGF research. We will address the concerns accordingly.

1. The authors used three strains belonging to three different genera for their proof of principal. This approach is good. My concern was with the choice of strains. The three genera are representatives of the three morphological varieties of AGF: filamentous monocentric, filamentous polycentric, and bulbous. Their spectra are expected to of course differ and the PCA is also expected to differentiate them into three clusters owing to their morphological differences. What if the same approach was used with three monocentric filamentous genera. Would the spectra be variable and would the PCA analysis be able to cluster them? If this is to be used on a larger scale beyond the proof of principal study, this has to be addressed. I understand that the authors might not have access to more strains due to the lack of a culture collection for AGF. Can they maybe add one more of each morphology to their experimental design. This will help tremendously.

We completely agree with the reviewer that the application of the techniques to a larger set of species would be helpful and that especially discrimination of morphologically similar and closely related strains would be interesting. We have also discussed this in more detail now in the manuscript (Discussion Lines 396-399 and Conclusion and Outlook Lines 604-611). As the reviewer has already mentioned, the largest challenge to this is the lack of available cultures of AGF and more importantly a lack of pure cultures, as AGF are mostly cultivated in co-culture with methanogens. The three strains used were the only ones accessible in pure culture in our lab. We initially also included Neocallimastix frontalis to have the precise comparison of two monocentric, filamentous strains (with Pecoramyces ruminantium). However, during the course of our experiments we discovered, that the culture was actually a co-culture with small amounts of methanogens. As we wanted to ensure that the chosen approach allowed for discrimination according to fungal biomass and not from the effects of presence/absence of other microorganisms we had excluded the Neocallimastix frontalis data from our overall analysis.

We have now included the initial testing of this strain in the discussion (Lines 419-426), as well as an additional supplementary figure, which displays the separation of the four strains in PCA analysis. While caution has to be taken due to the “contamination” with methanogens in the Neocallimastix frontalis culture, this displays the ability of the technique to discriminate morphologically similar strains.

2. I am still not clear on how this approach would be used for classification of pure cultures. Would one need to run strains of known identity each time an unknown sample is to be classified? If that is the case, then maybe more clarification on that can be added to the paper.

The currently developed model is able to discriminate the three selected strains. Unknown samples can only be correctly identified if they belong to a strain that has been used to create the model. Once a known strain is included in the model, it can be identified when presented as an unknown sample. We have added two sentences in Lines 429-432 to clarify this.

3. Also, the discussion on extending the use of this beyond pure cultures into fecal or rumen samples was not clear. Would the results from such analysis be similar to a culture-independent study and the components of the sample can be reported on as % abundances? More clarification is needed for that.

We have added additional description of possible applications for future HSI approaches in the Discussion section (Lines 581-586). We hope this makes our vision for application in environmental samples clearer.

The method could be used for culture-independent studies. As the identification is carried out with microscopy scanning, a precise percentage abundance could not be given, but estimates about how frequently certain species occur.

Otherwise, this was interesting and I would like to see the application on a wider scale in the AGF world.

Thank you for seeing the potential in our described method and sharing our vision for wider scale application in AGF research.

**Reviews of manuscript FEMSMC-2024-016.r1**

**Reviewer 1**

**Recommendation: Accept**

I have checked the revised manuscript and I can state that the authors responded to my comments and edited the text. I agree with the modifications and I recommend the article for publication.

**Reviewer 2**

**Recommendation: Accept**

Thank you for the revision. The authors addressed my concerns.

**Editor Decision: Accept**
